# SquiggleKit: a toolkit for manipulating nanopore signal data

**DOI:** 10.1093/bioinformatics/btz586

**Published:** 2019-07-23

**Authors:** James M Ferguson, Martin A Smith

**Affiliations:** Kinghorn Centre for Clinical Genomics, Garvan Institute of Medical Research, Darlinghurst, NSW, Australia; Kinghorn Centre for Clinical Genomics, Garvan Institute of Medical Research, Darlinghurst, NSW, Australia; St Vincent's Clinical School, Faculty of Medicine, UNSW Sydney, NSW, Australia

## Abstract

**Summary:**

The management of raw nanopore sequencing data poses a challenge that must be overcome to facilitate the creation of new bioinformatics algorithms predicated on signal analysis. SquiggleKit is a toolkit for manipulating and interrogating nanopore data that simplifies file handling, data extraction, visualization and signal processing.

**Availability and implementation:**

SquiggleKit is cross platform and freely available from GitHub at (https://github.com/Psy-Fer/SquiggleKit). Detailed documentation can be found at (https://psy-fer.github.io/SquiggleKitDocs/). All tools have been designed to operate in python 2.7+, with minimal additional libraries.

**Supplementary information:**

Supplementary data are available at *Bioinformatics* online.

## 1 Introduction

Nanopore sequencers, such as those manufactured by Oxford nanopore technologies (ONT), generate long sequencing reads by measuring disruptions in ionic current as biopolymers, such as DNA or RNA, transit through a nanopore (Garalde *et al.*, 2018; Laszlo *et al.*, 2014). The resulting signal is then de-convoluted into nucleotide sequence through probabilistic models, which often introduce errors given sampling stochasticity and imperfect models (Rang *et al.*, 2018; Schreiber and Karplus, 2015).

When a strand of nucleotides is sequenced with an ONT device, a data file is generated containing the raw signal and machine metadata. Sequencing runs can produce upwards of 100 million reads, which are grouped into a multitude of agglomerative binary files (.fast5). The yield of ONT sequencers is steadily increasing, and storing large amounts of data is resource intensive. Therefore, discarding the raw data once it has been converted into bases is a practical solution for many labs. However, the current base calling algorithms are limited in that they are error prone and do not include all known nucleotide analogues (Jonkhout *et al.*, 2017). This means that stored raw nanopore files can be revisited and mined to extract additional information as new and more advanced tools become available (Rand *et al.*, 2017; Simpson *et al.*, 2017).

In addition to storing the data for future use, users also need to process a vast quantity of files and extracting pertinent information from the binary file format can be a significant hurdle to overcome when analyzing nanopore data. Once the files are processed and the data are extracted, nanopore signal data remain difficult to navigate given the unfamiliar and noisy nature of single-molecule sensing. Altogether, these challenges hinder the full utilization of nanopore data, the development of new bioinformatics tools and the training of more accurate machine learning algorithms.

Although existing software applications contain methods for file management, data extraction, plotting, segmentation and signal to base comparison, none encapsulate all of these applications (a detailed comparison of these tools is presented in the Supplementary Material). Furthermore, some of these tools are outdated, unsupported, or are limited to specific use cases, such as modification detection or real-time analysis, highlighting the need for a modular, general use toolkit for nanopore signal data mining.

Here, we describe SquiggleKit, a unified toolkit for extracting, processing and plotting raw nanopore sequencing signal data. In addition to data mining applications, SquiggleKit can be used to manage the extensive number of data files generated during nanopore sequencing, as well as a starting point for developing new tools predicated on nanopore signal data.

## 2 The toolkit

- *File management and processing:* **Fast5_fetcher** extracts individual fast5 files from an index based on a list of reads of interest, thus reducing both search time and storage space. **SquigglePull** opens fast5 files, extracts the embedded signal data and converts it into a tab separated (.tsv) format.
- *Visualization*: **SquigglePlot** is a command line visualization tool for signal data.
- *Targeting regions of interest in raw signal data*: **Segmenter** identifies the boundaries of relatively long regions of signal attenuation, such as adapter stalls and homopolymer stretches, by measuring the difference in the average signal over a minimal distance, with error tolerance for signal noise. **MotifSeq** identifies raw signal traces that correspond to a given nucleotide sequence, such as an adapter, barcode, or motif of interest. MotifSeq takes a query nucleotide sequence as input, converts it to a normalized signal trace (i.e. ‘events’), then performs signal-level local alignment using a dynamic programming algorithm. MotifSeq outputs the location of a matching target in the raw signal with an associated *P*-value (Supplementary Material).

## 3 Practical example

SquiggleKit can be used to facilitate data management, to generate fine-tuned datasets for machine learning, to visualize signal, to validate de-multiplexing results and to identify motifs of interest without base calling, amongst other applications.

**Fig. 1.**
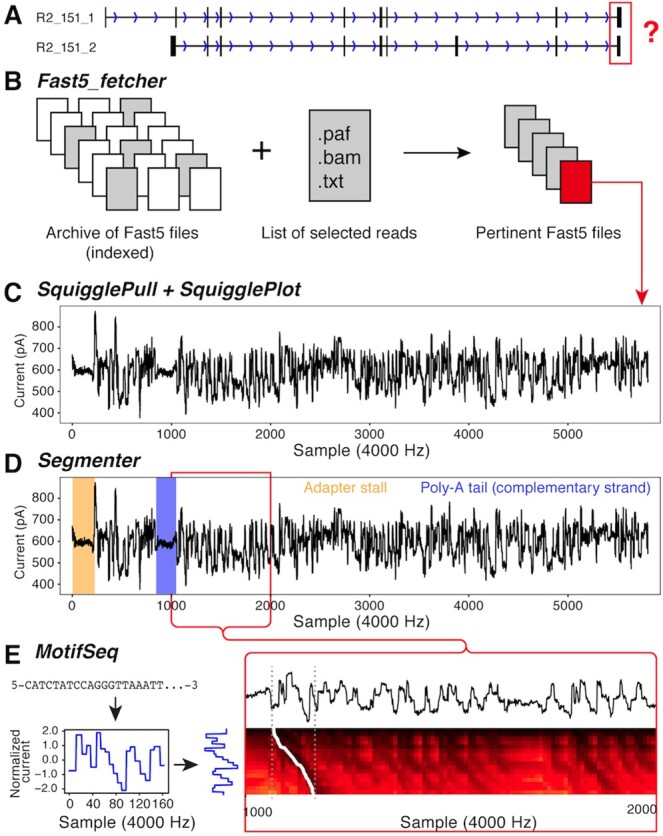
(**A**) Two similar reference transcripts with similar mapping. (**B**) Using fast5_fetcher to extract raw files. (**C**) SquigglePull and SquigglePlot converting to.tsv and plotting the signal. (**D**) Adapter stalls and poly-A tail identified using Segmenter. (**E**) Converting last 20 nt of the 3’ end to a signal using a pore current model and aligning this synthetic signal to the empirical signal (dashed grey lines) by backtracking through a dynamic programming matrix (white trace)

In the following example, we demonstrate how SquiggleKit can be used to validate the 3’ end of a terminal exon using raw nanopore signal data from a cDNA run. Specifically, a cDNA read that aligned to two distinct isoforms (A and B) from a reference transcriptome (Fig. 1A). We will interrogate the raw signal to identify the 3’ end of one of the reference isoforms.

First, a list of all read identifiers that mapped to isoform A is extracted from the alignment output into a .txt file. This list is used as input for Fast5_fetcher (Fig. 1B) together with an index of the archived raw data files (.fast5.tar). Fast5_fetcher outputs a subset of extracted .fast5 files instead of all 2 710 372 files that are present in the full dataset. This decreases computational load and processing times of the subsequent steps.

Next, the raw ionic signal for the read of interest is extracted with SquigglePull and plotted with SquigglePlot (Fig. 1C). The .tsv output of SquigglePull is then used as input for Segmenter to identify the adapter stall and poly-A homopolymer sequence (Fig. 1D). The raw signal directly downstream of the poly-A signal is then extracted with Segmenter, thus selecting the 3’ end of the terminal exon in the signal data.

Finally, the nucleotide sequence corresponding to the last 20 nucleotides of the 3’ end of isoform A is converted into normalized signal space. The resulting theoretical current trace is aligned to the empirical signal from Segmenter using MotifSeq (Fig. 1E), returning the position of the sequence in the terminus of the raw signal, a match score of 39.70 and associated *P*-value of 0.0169 (*z*-test), thus confirming the exon boundary.

## 4 Conclusion

SquiggleKit helps researchers extract more information from their data by making raw signal analysis more accessible. We anticipate that this toolkit will facilitate the development of future bioinformatics tools and help create more accurate probabilistic models for nanopore sequencing data analysis, in a timely and user friendly manner.

## Supplementary Material

btz586_Supplementary_Data
