# Supplementary material for: SquiggleKit: a toolkit for manipulating nanopore signal data
Source: Bioinformatics. 2019 Jul 23;35(24):5372–3. doi: 10.1093/bioinformatics/btz586 (PMC9887521; doi:10.1093/bioinformatics/btz586)
Supplement: btz586_Supplementary_Data [file bioinformatics_35_24_5372_s2.pdf]

SquiggleKit: supplementary information

Contents:

[Contents:](#)

[1. Detailed description of finding 3’ end motif example](#)

[2. Comparison to accessible methods with related functions](#)
- [Table 1: Overview of tool comparisons](#)

[3. Extended comparison](#)
- [BulkVis](#)
- [Poretools](#)
- [Picopore](#)
- [PoRe](#)
- [HDFView](#)
- [Japsa](#)
- [ReadUntil](#)
- [Tombo/NanoRaw](#)
- [Nanopolish](#)

[4. Standardisation of signal for comparison](#)
- [Sup. Figure 1: Comparison of raw signal scaling strategies on MotifSeq scoring](#)

[5. Benchmarking](#)
- [System specifications:](#)
- [Fast5_fetcher speed](#)
- [Table 2: Fast5_fetcher speed benchmark](#)
- [Segmenter speed](#)
- [Table 3: Segmenter speed (default parameters on cDNA sequins)](#)
- [MotifSeq speed](#)
- [Table 4: MotifSeq speed in function of k-mer length (default parameters on .tsv files)](#)
- [Segmenter accuracy](#)
- [Sup. Figure 2: Segmenter boundary assessment](#)
- [Table 5: Segmenter Accuracy](#)
- [MotifSeq accuracy](#)
- [Sup. Figure 3: MotifSeq scoring benchmark](#)

[6. Supplementary References](#)

1. Detailed description of finding 3' end motif example

Note: The use of Parallel Alignment File (PAF) format over sam format is arbitrary. It does however only keep reads that aligned, whereas sam includes all reads, thus streamlining fast5 filtering.

1. Aligning reads with *minimap2* with paf output:

```
$ minimap2 -x ont-map sequin.fa reads.fastq > alignment.paf
```

2. Filtering paf file for reads of interest, where R2_151 represents a unique identifier for the transcript group of interest.

```
$ grep R2_151 alignment.paf > filtered.paf
```

3. Using a filtered .paf file (generated in 2.) as input for *fast5_fetcher*:

```
$ mkdir fast5
$ python fast5_fetcher.py -p filtered.paf -s sequencing_summary.txt
-i name.index -o ./fast5
```

4. Extract signals from fast5 files to a tab separated values (tsv) file:

```
$ python SquigglePull.py -rv -p ./fast5/ -f all > signals.tsv
```

5. Plot signals:

```
$ python SquigglePlot.py -s signals.tsv --save R2_151.pdf --
save_path ./pics/ --no_show
```

6. Identify any segments in signals and visualise each one:

```
$ python segmenter.py -s signals.tsv -v > segments.tsv
```

7. Motif identification. Fasta format for creating signal from model (in a custom file named *motif.fa* for this example):

```
>my_kmer_name
CATCTATCCAGGGTTAAATT
```

8. Find the best match to that k-mer in the signal, and visualise it:

```
$ python MotifSeq.py -s signals.tsv -i motif.fa -v >
signals_kmer.tsv
```

2. Comparison to accessible methods with related functions

Table 1: Overview of tool comparisons

Tools	*SquiggleKit*				
	fast5_fetcher	*SquigglePull*	*SquigglePlot*	*Segmenter*	*MotifSeq*
BulkVis					
Poretools					
Picopore					
poRe					
HDFView					
Japsa					
ReadUntil					
Tombo/NanoRaw					
Nanopolish					

Legend

Not the same	Similar functionality	Equal functionality

Extraction and Visualisation:

- *Poretools* - extract events, not raw (Loman and Quinlan, 2014)
- *poRe* - extract events, not raw (Watson *et al.*, 2015)
- *Nanoraw* - raw signal visualisation and comparison, aligns signal to base
- *Tombo* - raw signal visualisation and comparison, aligns signal to base (Stoiber *et al.*, 2017)
- *HDFView* - single file, basic visualisation (http://www.hdfgroup.org/HDF5/)
- *BulkVis* - bulk fast5 files, real time (Payne *et al.*, 2018)

File Management:

- *Picopore* - removes data from fast5 files to reduce size (Gigante, 2017)
- *Japsa* - manages files, streams data and performs base space analysis (Nguyen *et al.*, 2017)

Analysis:

- *ReadUntil* - local dynamic programming, real time (Loose *et al.*, 2016)
- *Nanopolish* - File management, segmentation, aligns signal to base (Loose *et al.*, 2016; Loman *et al.*, 2015; Loman and Quinlan, 2014)

3. Extended comparison

**Comparison**	**BulkVis**
Info	*BulkVis* enables visualization of bulk FAST5 files collected from Nanopore sequencers.<br>*BulkVis* is provided for the visual inspection of challenging or difficult to sequence samples or where the user wishes to investigate specific events during a run. In these instances analysis of a bulk FAST5 file may provide some visual indication of the underlying issues
Last update	September 2018
Links	https://academic.oup.com/bioinformatics/advance-article/doi/10.1093/bioinformatics/bty841/5193712<br>https://bulkvis.readthedocs.io/en/latest/index.html
Similarities	Parses sequencing_summary.txt, .paf, and bulk fast5 files to break down MinKNOW states: above, adapter, pore, transition, unblocking, and unclassified. Outputs in a .csv. This is similar to the adapter stall targeting by segmenter, however the segmenter algorithm is novel.<br><br>It allows for great visualisation and annotation of signals in a bulk fast5 file. This is similar to *SquigglePlot*, but are used for different applications
Differences	*SquigglePlot* and segmenter work with regular fast5 files, or any signal input, where *BulkVis* plots bulk fast5 files.
Comments and use	It has a specific use case around examining reads using MinKNOW's classifications and requires bulk fast5 file.

**Comparison**	**Poretools**
Info	*Poretools* provides a wealth of utilities and data exploration tools.

Last update	10 Jul 2017
Links	https://github.com/arq5x/poretools
Similarities	*Poretools* contains event data extraction similar to that of *SquigglePull*
Differences	*Poretools* extracts event data, which is no longer used as the basis of basecalling.<br>Events changed to a static time for sampling and changed in format, breaking Poretools for the latest data.
Comments and use	*Poretools* was one of the first tools used for generating stats and getting a feel for nanopore data. It is no longer routinely used, and does not have support for raw signal data.<br>If the user is after raw signal data and visualisation, picking *SquigglePull* and *SquigglePlot* would be the superior choice

**Comparison**	**Picopore**
Info	A tool for reducing the size of Oxford Nanopore Technologies' datasets without losing information.
Last update	31 Aug 2017
Links	http://dx.doi.org/10.12688/f1000research.11022.1<br>https://github.com/scottgigante/picopore
Similarities	Reduction in memory footprint, but by gutting fast5 files of various information. This is similar to extracting the raw signal with *SquigglePull* and gzipping.<br>The reduction in memory footprint also overlaps with the reasoning behind *fast5_fetcher*, downsizing to only mapped fast5 files, or fast5 files of interest
Differences	The methodology of memory footprint for storage or use are quite different, where *picopore* guts and compresses data, *fast5_fetcher* filters and *SquigglePull* extracts into a different format.
Comments and use	*Picopore* is no longer under active development. Due to improvements in ONT's native HDF5 compression, lossless and deep-lossless compression no longer effectively reduce the size of nanopore files.<br>With the increase in yields, and lack of methods to compress that data, both *fast5_fetcher* and *SquigglePull* can be of use to reduce

	what data you need depending on the kind of analysis you are trying to undertake.

**Comparison**	**PoRe**
Info	This package enables organisation and visualisation of MinION data on the MinION laptop for the novice user.
Last update	31 Aug 2017
Links	https://github.com/mw55309/poRe_docs<br>https://sourceforge.net/projects/rpore/files/<br>https://github.com/mw55309/poRe_scripts
Similarities	*poRe* contains event data extraction similar to that of *SquigglePull*
Differences	*poRe* does not extract or visualise raw signal
Comments and use	*poRe* is no longer under development. It works with legacy fast5 files and older basecaller data structures.

**Comparison**	**HDFView**
Info	*HDFView* is a visual tool written in Java for browsing and editing HDF5 files. View a file hierarchy in a tree structure. Create new files, add or delete groups and datasets. View and modify the content of a dataset. Add, delete and modify attributes.
Last update	updated current 2019
Links	http://www.hdfgroup.org/HDF5/
Similarities	*HDFView* allows for basic visualisation of raw signal data, with overlap with *SquigglePlot*
Differences	*SquigglePlot* has many more features than HDFView for visualisation, and can plot many files at once with custom settings, at high DPI for figures.
Comments and use	*HDFView* is excellent for exploring a fast5 file for information and structure or visualising one signal.<br>When working with many signals, or just wanting to look at the signal directly, *SquigglePlot* is superior.

Comparison	Japsa
Info	*Japsa* has many packages that do a variety of nanopore data analysis and data streaming.
Last update	update current 2019 - multi package project
Links	https://japsa.readthedocs.io/en/latest/index.html<br>https://www.ncbi.nlm.nih.gov/pubmed/28961965
Similarities	*Japsa* has packages for real time file management and analysis during sequencing.
Differences	*Japsa* employs many base space sequence analysis methods like barcode demultiplexing. It does not however do any analysis, extraction, or visualisation of raw signal
Comments and use	*Japsa* seems to have been built as a pipeline for many real time analysis methods. It does not have any significant overlap other than file some file management.

Comparison	ReadUntil
Info	*ReadUntil* has a function, squiggle_search2(), to find if a particular sequencing matches a selected region or not, using the coordinates/position of the match. This is specifically designed for *ReadUntil*, and the scripts and methods can not be used for general exploration.<br>No plotting of raw signal.
Last update	2016
Links	https://www.nature.com/articles/nmeth.3930<br>http://mattloose.github.io/RUscriptsdocs/<br>https://github.com/mattloose/RUscripts/tree/master/ReadUntil
Similarities	*ReadUntil* takes a reference sequence region and converts it into the event space using the models for basecalling. It then uses dynamic programming on the first portion of an incoming read to test if it matches within the selected region.
Differences	*MotifSeq* is a general method for finding the approximate position of where a particular sequence motif aligns with the raw signal data.

	ReadUntil and *MotifSeq* do this is similar ways, however while *ReadUntil* does the comparisons in event space with streaming data, *MotifSeq* does the comparisons in raw signal space.
Comments and use	squiggle_search2() from *ReadUntil* finds if a particular sequence matches a selected region or not, using the coordinates/position of the match. This is specifically designed for *ReadUntil*, and the scripts and methods can not be used for general exploration as is.

**Comparison**	**Tombo/NanoRaw**
Info	*Nanoraw* and *tombo* operate based on the genome_resquiggle/re-squiggle method, mapping the raw signal with basecalls and reference alignment. All visualisation is centred around this method, and though it has the potential to be general use, it is not implemented or designed that way.
Last update	last updated current in 2019
Links	https://www.biorxiv.org/content/biorxiv/early/2017/04/10/094672.full.pdf<br>https://nanoporetech.github.io/tombo/tutorials.html<br>https://nanoraw.readthedocs.io/en/latest/resquiggle.html
Similarities	*Tombo* can visualise raw signal data similar to *SquigglePlot*.<br>*Tombo* can centre the visualisation around a particular motif, and extract that information with the API to find the signal associated with the basecalls, similar to *MotifSeq*.<br>*Tombo* can extract the signal data, similar to *SquigglePull*.
Differences	*Tombo* can visualise multiple squiggles on one plot, and can associate them with the basecall as well creating a squiggle pileup plot.<br>While *Tombo* focuses on multiple squiggles, *SquigglePlot* focuses on one squiggle at a time.<br>*Tombo* uses the resquiggle algorithm to match the aligned base sequence to the raw signal.<br>*MotifSeq* converts the sequence motif to a signal using a model, and uses a local dynamic programming method to identify the position of the motif.<br>The signal extraction in *SquigglePull* is more general than tombo's pipeline.

Comments and use	*Tombo* is a fully fledged toolkit for finding differences in signal space, and exploring the raw signal of a dataset.<br>*Tombo* and *NanoRaw* were designed to find differences in the signal to identify modifications, and help train models to detect them.<br>*Fast5_fetcher* may be of use for filtering large datasets to be analysed with *tombo*<br>Use of either toolkit would be based on specific goals

**Comparison**	**Nanopolish**
Info	*Nanopolish* is a software package for signal-level analysis of Oxford Nanopore sequencing data. *Nanopolish* can calculate an improved consensus sequence for a draft genome assembly, detect base modifications, call SNPs and indels with respect to a reference genome and more.
Last update	current 2019
Links	https://github.com/jts/nanopolish<br>https://nanopolish.readthedocs.io/en/latest/index.html
Similarities	File management, segmentation, aligns signal to base are similar to *fast5_fetcher*, *segmenter*, and *MotifSeq* respectively.
Differences	*Nanopolish* is aimed at specific problems and the tool is created as an integrated method of analysis, where *SquiggleKit* tools can all work standalone and in a more general way.
Comments and use	*Nanopolish* is best suited to standard methods of analysis.<br>*SquiggleKit* is best used for developing tools like *nanopolish*, or exploring nanopore signal data.

4. Standardisation of signal for comparison

Many factors can contribute to non-sequence dependent variability in nanopore sequencing data (temperature, voltage, etc), which can lead to progressive distortion of raw current values for a single read. The three main types of raw signal distortion are shift, scale, and drift, which can be mitigated by normalising or standardising raw signal using global properties of the data. Standardisation is required to compare 2 raw signals against each other, or a simulated signal from the basecalling model and a raw signal, as implemented in *MotifSeq*.

We compared 3 methods for raw signal normalisation: Z-score scaling, Median-Median-Absolute-Difference scaling (med-MAD), and pico-Ampere scaling.

Z-score scaling is performed using the function **z = (x - u) / s**, where **u** and **s** are the mean and standard deviation of the signal, respectively. It is more sensitive to any large spikes in the signal (which are relatively common), thus impacting the overall mean and standard deviation. One method of offsetting this is to filter large spikes above and below a threshold, as is done with the --scale_hi/--scale_low flags in *MotifSeq*, set at 900 and 0 respectively for DNA.

Alternatively, raw signal can be shifted and scaled by converting to pico ampres, then scaled. This is done by extracting the digitisation, offset, and range values from the fast5 files, and converting the raw data points using the function **pA = (x + offset) * (range/digitisation)**. This can be done automatically when the raw signal is extracted using the --convert flag in *SquigglePull*. There is no significant difference in *MotifSeq* scores when using Z-score or Z-score pA scaling (**Supplementary Figure 1C**).

MotifSeq can do Z-score or median-absolute-difference scaling, in a similar way, but which are more or less sensitive to large spikes in the signal. The med-MAD scaling uses the following function: **z = (x - med) / MAD**, where **med** and **MAD** are the median and Median-Absolute-Difference of the signal respectively. Med-MAD is more robust at handling noise in nanopore signals than z-score scaling, where the mean and standard deviation can be significantly impacted by the occasional spike in current. Med-MAD is used by nanopore basecalling software for this reason. This is evidenced in **Supplementary Figure 1A-B** by slightly lower MotifSeq scores, indicative of lower penalties for outlier spikes in current.

All 3 normalisation methods have been implemented in MotifSeq, which uses med-MAD by default given the benchmarking results described below (see section 5).

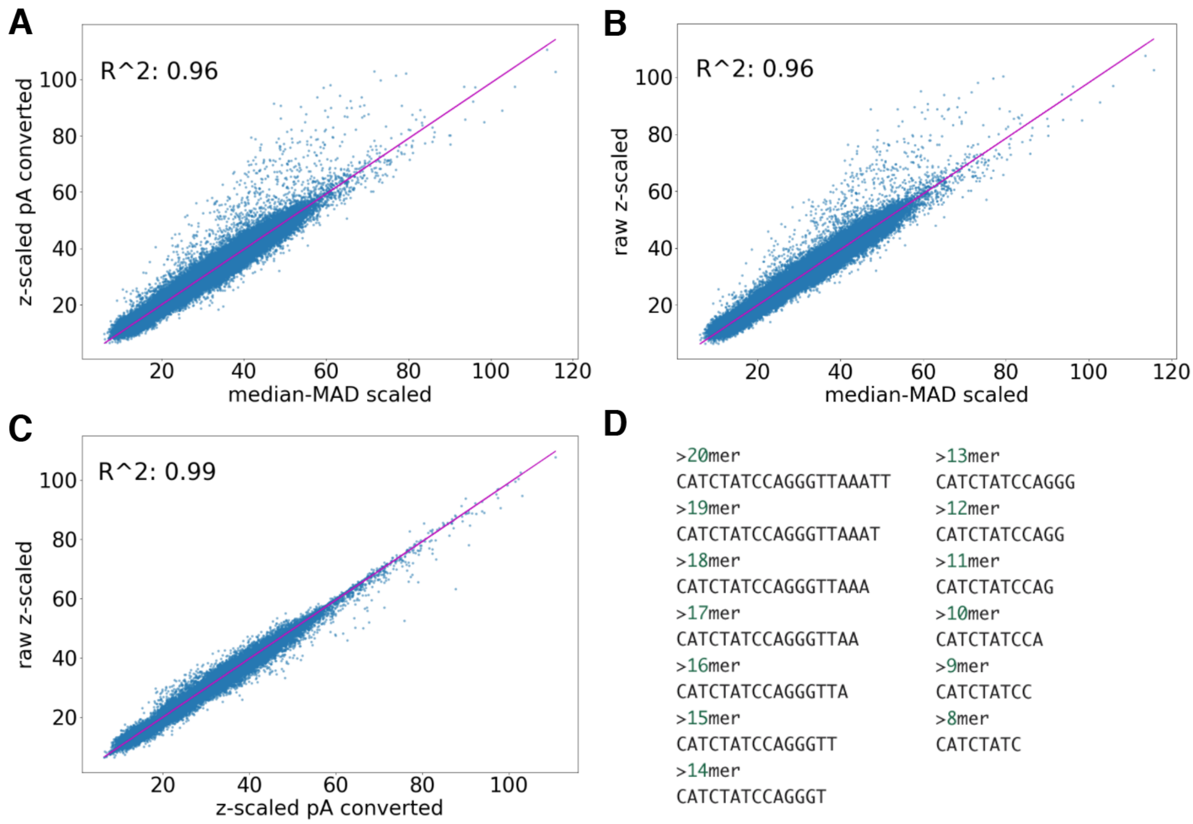

Sup. Figure 1: Comparison of raw signal scaling strategies on MotifSeq scoring

(**A-C**) Pairwise comparison of MotifSeq scores for all combinations of raw Z-score scaling (raw z-scaled), raw Z-score scaling with conversion to picoAmperes (z-scaled pA converted), and median-median-absolute-difference (median-MAD scaled). Scores were generated by running MotifSeq with 12 different k-mers (**D**) corresponding to the 3' end used in the main example against the raw signals of 10,000 randomly selected cDNA reads, which were scaled using either of the 3 methods described above.

5. Benchmarking

Benchmarking was carried out to assess speed and accuracy for certain *SquiggleKit* tools. Below is a comparison of speed for *fast5_fetcher*, *segmenter*, and *MotifSeq*. Accuracy benchmarking was performed for *Segmenter* and *MotifSeq*. All local benchmarks were carried out on the same hardware, as described below.

System specifications:

Linux omega-1-31.local 2.6.32-642.3.1.el6.x86_64
x86_64 x86_64 x86_64 GNU/Linux
CPU: AMD Opteron(tm) Processor 6282 SE (only 1 CPU used)
CPU MHz: 2600
Architecture: x86_64
RAM: 32GB
Disk: 400GB NVME
Python version: 2.7.14

Fast5_fetcher speed

By taking a dataset with 7,332,202 single fast5 files, tarballed into groups of 4000, the average runtime (after 5 replicates) of *fast5_fetcher* extraction was measured at different magnitudes (i.e. number of files to extract).

Table 2: Fast5_fetcher speed benchmark

Number of files	Size of files	Average runtime (s)	Reads per Second
1,000	32MB	204.2	4.9
10,000	697MB	1673.8	6.0
100,000	9.7GB	20133.8	5.0
1,000,000	102GB	259546	3.9

Segmenter speed

S*egmenter* identifies regions of low complexity using a greedy algorithm in a manner similar to a Markov chain. It processes raw signal as follows:

1. Calculate the median current intensity of the full read;
2. Set thresholds about the median using a fraction of the standard deviation (parameter --std_scale, default 0.75);
3. For each data point, calculate the difference to the median. Stretches of signal are returned if at least *w* (parameter --window, default 150) consecutive data points are within the standard deviation threshold;
4. Outliers are tolerated (parameter --error, default 5) as are consecutive low complexity regions within a given boundary (parameter --seg_dist, default 50).

Table 3: Segmenter speed (default parameters on cDNA sequins)

Number of files	time (s)	Reads per second	Time for gzip input (s)	Reads per second (gzip)
103,526	916	~113	3,430	~30

MotifSeq speed

Table 4: MotifSeq speed in function of k-mer length (default parameters on .tsv files)

K-mer length	Total time (s)	Reads per second	Time per read (s)
12	92.13	54.27	0.0184
30	143.82	34.77	0.0288
50	232.05	21.55	0.0464
75	338.47	14.77	0.067
100	402.98	12.41	0.080
256	963.55	5.19	0.193

This is comparable to the speed attained by (Loose *et al.*, 2016) in their speed testing on "Intel(R) Xeon(R) E5-2690 version 3 central processing units (CPUs) running at 2.60 GHz (server)" attaining ~0.3 seconds per read for a k-mer length of 256.

Segmenter accuracy

Segmenter is designed to identify low complexity regions in raw nanopore signal above a given window length. It was designed as a qualitative annotation tool to identify homopolymer boundaries and polymer translocation stalling. Although it could be used to find associations between poly-A tail lengths and signal length, this is not the subject of this toolkit.

Identifying low complexity regions in raw signal is not straightforward to benchmark given the stochastic nature of single molecule sensing. Consequently, we evaluated the accuracy of *Segmenter* by manually inspecting 100 raw cDNA signals using *Segmenter*'s "-v" argument (**Supplementary Figure 2**). Homopolymer boundaries corresponding to poly-A regions in cDNA reads were visually identified, their position recorded and compared to the those automatically identified by *Segmenter* (**Table 5**).

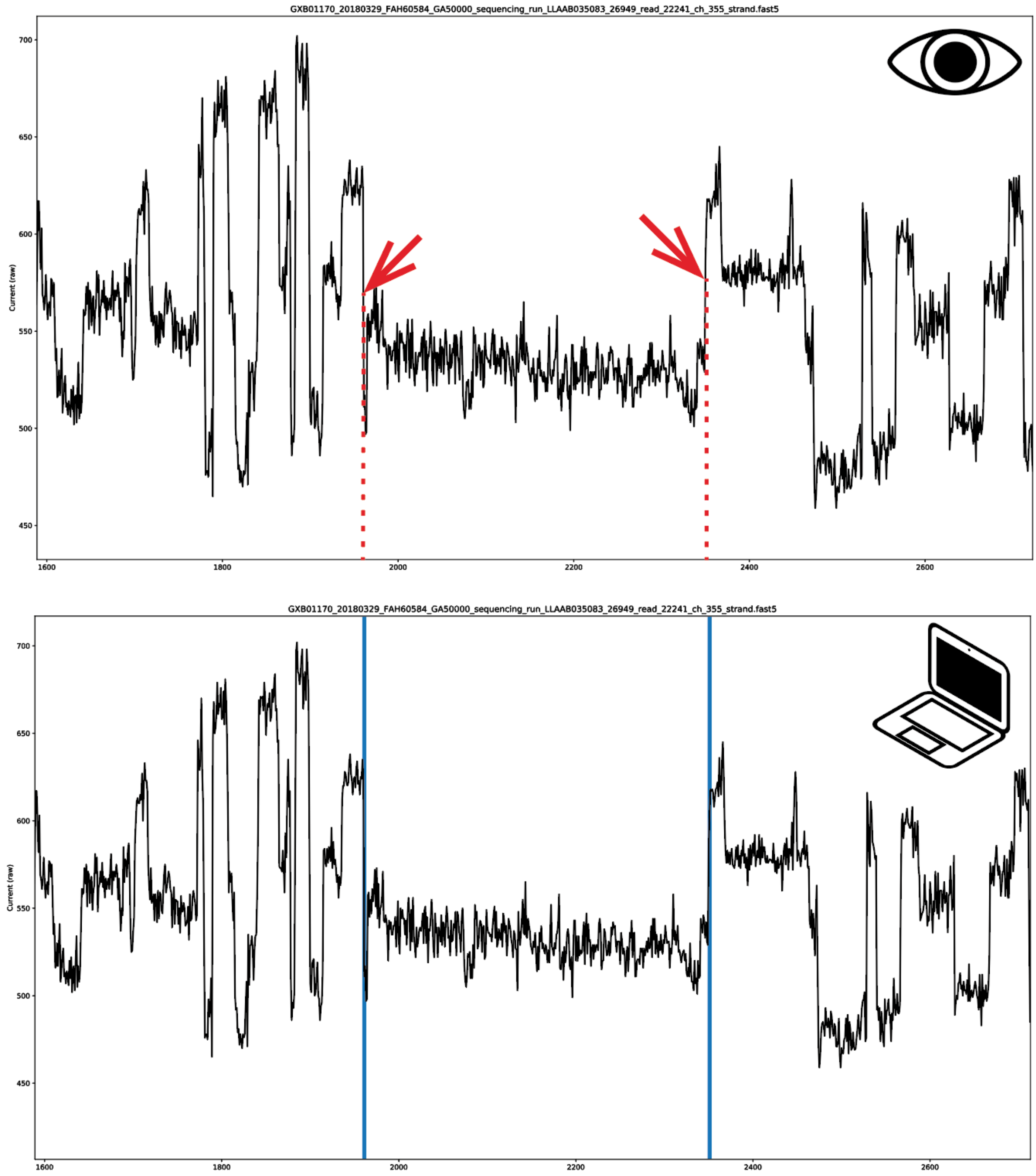

Sup. Figure 2: Segmenter boundary assessment

Low complexity regions identified by *Segmenter* were manually inspected (top) to identify precise boundaries visually using *matplotlib* functionality. The positions on the x-axis corresponding to boundary signals (red lines) were recorded and compared to those output by *Segmenter* (bottom, blue lines),

Table 5: Segmenter Accuracy

Differences	Mean difference	Median difference	Stdev of difference
Start	2.32	1.0	5.4
End	9.25	3.0	13.7
Length	9.75	3.0	14.7

As can be seen, start accuracy is quite good, with the largest variation in the end position targeting. A mean length difference of 9.75 data points approximately corresponds to 1 nucleotide worth of signal (~8-11 data points per nucleotide for DNA pore models).

MotifSeq accuracy

We benchmarked *MotifSeq* accuracy by comparing minimum dynamic programming scores across positive and negative control datasets, which were generated as follows:
(i) Using a sequin synthetic RNA spike-in control cDNA sequencing run (ENA accession number PRJEB33439), we randomly selected a sequin isoform from all isoforms with at least 10,000 uniquely mapping reads (minimap2 using the preset "-x map-ont") and a MAPQ score of 60 via the resulting .paf file.
(ii) The corresponding reference transcript (available at www.sequin.xyz) was used to randomly extract k-mers (within 15nt of the transcript extremities) of lengths 12, 30, 50, 75, and 100 nucleotides.
(iii) For each k-mer, positive and negative control sets were generated by identifying all reference sequin isoforms with or without the selected k-mer, respectively, using direct sequence matching.
(iv) A random selection of 5000 cDNA reads were selected that uniquely map to the reference isoforms for both control sets defined in (iii). We ensure the mapped sequences overlapped the position of the k-mer identified in the reference sequences. Reads were selected with *fast5_fetcher* and raw signals extracted using *SquigglePull*.
(v) Each k-mer motif was submitted to *MotifSeq* comparisons across both positive and negative control datasets (**Supplementary Figure 3**).

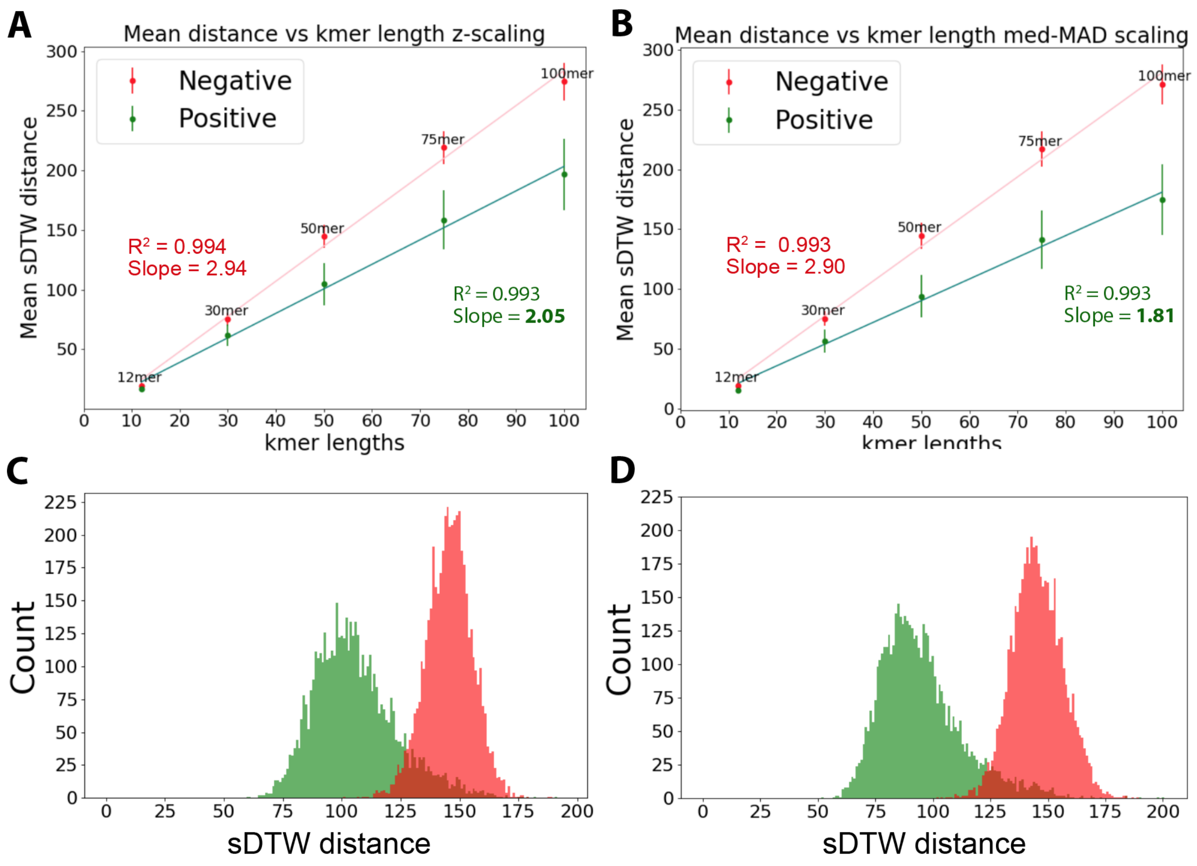

Sup. Figure 3: MotifSeq scoring benchmark

The *MotifSeq* mean score (sDTW distance) and standard deviation (error bars) for 5000 comparisons against positive (red, top) and negative (green, bottom) controls is plotted for 5 different k-mer sizes in conjunction with (**A**) z-scaling and (**B**) med-MAD scaling of raw signal. A linear regression trend line is fitted to 5 different k-mer sizes with associated slopes and correlation of determination (R^2). (**C**) Distribution of *MotifSeq* scores for 50-mers using z-scaling and (**D**) med-MAD scaling.

As expected, the separation of true positive scores from true negative scores increases with k-mer length. The separation further increases when using med-MAD scaling vs z-scaling, highlighting the improved tolerance of med-MAD scaling for outlier current spikes through slightly lower scores overall. *MotifSeq* scores against the negative control appear to be normally distributed (**Supplementary Figure 3C-D**, in red or on the right) suggesting that they can be interpreted as Z-scores to assess the confidence of hits via the background mean and standard deviation, which can be converted to a probability using the formula:

$$P(X < \mu) = P\left(\frac{X - \mu}{\sigma} < \frac{\mu_q - \mu_b}{\sigma_b}\right)$$

Where $\boldsymbol{\mu}$ is the mean, μ_q is the query mean, μ_b is the background mean, $\boldsymbol{\sigma}_b$ is background standard deviation.

Furthermore, the seemingly linear correlation between *MotifSeq* scores and k-mer lengths suggests that linear regression can be used to extrapolate background models to a given motif size (**Table 6**). Consequently, we incorporated this additional scoring metric into *MotifSeq*, which now

Table 6: Expected background mean and standard deviation by k-mer length

k-mer	Mean	stdev	k-mer	Mean	stdev
**10**	19.44	1.65	**160**	455.16	38.54
**15**	33.96	2.88	**165**	469.68	39.77
**20**	48.49	4.11	**170**	484.21	41
**25**	63.01	5.34	**175**	498.73	42.23
**30**	77.54	6.57	**180**	513.26	43.46
**35**	92.06	7.8	**185**	527.78	44.69
**40**	106.58	9.03	**190**	542.3	45.92
**45**	121.11	10.26	**195**	556.83	47.15
**50**	135.63	11.49	**200**	571.35	48.38
**55**	150.16	12.72	**205**	585.87	49.61
**60**	164.68	13.95	**210**	600.4	50.84
**65**	179.2	15.18	**215**	614.92	52.07
**70**	193.73	16.4	**220**	629.45	53.3
**75**	208.25	17.63	**225**	643.97	54.53
**80**	222.78	18.86	**230**	658.49	55.76
**85**	237.3	20.09	**235**	673.02	56.99
**90**	251.82	21.32	**240**	687.54	58.22
**95**	266.35	22.55	**245**	702.07	59.45
**100**	280.87	23.78	**250**	716.59	60.68
**105**	295.4	25.01	**255**	731.11	61.91
**110**	309.92	26.24	**260**	745.64	63.14
**115**	324.44	27.47	**265**	760.16	64.37
**120**	338.97	28.7	**270**	774.69	65.6
**125**	353.49	29.93	**275**	789.21	66.83
**130**	368.02	31.16	**280**	803.73	68.06
**135**	382.54	32.39	**285**	818.26	69.29
**140**	397.06	33.62	**290**	832.78	70.52
**145**	411.59	34.85	**295**	847.31	71.75
**150**	426.11	36.08	**300**	861.83	72.98

**155**	440.64	37.31			

**Median-MAD scaling was used for modelling.*

6. Supplementary References

Gigante,S. (2017) Picopore: A tool for reducing the storage size of Oxford Nanopore Technologies datasets without loss of functionality. *F1000Res.*, **6**, 227.

Loman,N.J. *et al.* (2015) A complete bacterial genome assembled *de novo* using only nanopore sequencing data. *Nat. Methods*, **12**, 733.

Loman,N.J. and Quinlan,A.R. (2014) Poretools: a toolkit for analyzing nanopore sequence data. *Bioinformatics*, **30**, 3399–3401.

Loose,M. *et al.* (2016) Real-time selective sequencing using nanopore technology. *Nat. Methods*, **13**, 751.

Nguyen,S.H. *et al.* (2017) Real-time demultiplexing Nanopore barcoded sequencing data with npBarcode. *Bioinformatics*, **33**, 3988–3990.

Payne,A. *et al.* (2018) BulkVis: a graphical viewer for Oxford nanopore bulk FAST5 files. *Bioinformatics*.

Stoiber, M.H. (2017) De novo Identification of DNA Modifications Enabled by Genome-Guided Nanopore Signal Processing, bioRxiv, https://doi.org/10.1101/094672 .

Watson,M. *et al.* (2015) poRe: an R package for the visualization and analysis of nanopore sequencing data. *Bioinformatics*, **31**, 114–115.
